# Prevalence and associated risk factors among patients with overactive bladder syndrome in Pakistan

**DOI:** 10.12669/pjms.37.4.4262

**Published:** 2021

**Authors:** Sajid Rashid, Muhammad Naveed Babur, Rehan Ramzan Khan, Muhammad Usman Khalid, Haroon Mansha, Saima Riaz

**Affiliations:** 1 Prof. Dr. Sajid Rashid, PP-DPT. Principal / HOD Multan College of Physiotherapy, MMDC, Multan, Pakistan; 2 Prof. Dr. Muhammad Naveed Babur, PhD. Principal, Isra Institute of Rehabilitation Sciences, Isra University, Islamabad, Pakistan; 3 Dr. Rehan Ramzan Khan, MSPT-OMPT. Assistant Professor Multan College of Physiotherapy, MMDC, Multan, Pakistan; 4 Dr. Muhammad Usman Khalid, MSPT-OMPT. Assistant Professor Multan College of Physiotherapy, MMDC, Multan, Pakistan; 5 Dr. Haroon Mansha, tDPT. Assistant Professor Multan College of Physiotherapy, MMDC, Multan, Pakistan; 6 Dr. Saima Riaz, PhD. Assistant Professor Riphah College of Rehabilitation & Allied Health Sciences, RIU, Lahore, Pakistan

**Keywords:** Overactive bladder, OABSS, Prevalence, Risk factor

## Abstract

**Objectives:**

To determine the Prevalence and associated risk factors among patients with overactive bladder syndrome in Pakistan.

**Methods:**

This was a community-based, face to face, cross sectional survey to calculate the prevalence and its associated risk factors. A sample of 1058 patients, women and men aged between 35 to 60 years having symptoms of overactive bladder was selected through convenience sampling from different cities of Pakistan during September to December 2020. Data was collected by using an Overactive Bladder Scoring System (OABSS) tool for prevalence and a developed questionnaire to rule out the risk factors.

**Results:**

The prevalence was 27.4% (n=289) and it increased with age. The average ages for women and men were 44.60±7.88 and 46.14±7.69 years respectively. The OAB prevalence was the lowest among the participants aged 35-43 years 15.2% (n=55) while it was highest among those who were aged 53-60 years 49.6%, (n=127). The age, body mass index, diabetes mellitus, income, family history, parity and urinary tract infection were found to be significant associated risk factors for overactive bladder with p value <0.05.

**Conclusion:**

The overall prevalence of overactive bladder was 27.4% and it does not differ by gender, hypertension, pelvic surgery, smoking, constipation and sleep while it has significant association with age, body mass index, diabetes mellitus, income, parity and urinary tract infections.

## INTRODUCTION

The overactive bladder (OAB) is a common disorder which affects numerous human lives in the world.^1^ In spite of the fact that OAB is curable, yet most of the patients of OAB are unaware of it and they do not try to get medical attentions. Lower urinary tract symptoms including micturition, urgency with or without incontinence, nocturia and polyuria cause overactive bladder.^2^ In diagnosing OAB, urgency is considered to be the major symptom. It is related to depression in the elderly population; decreased health related quality of life and decreased sleep quality.^1^,^3^ The overactive bladder results in emotional, social, physical, sexual and psychological problems and it is thought to be a serious health issue. Many factors including alcohol consumption, parity, sex, body mass index (BMI), age, hypertension, race, constipation, smoking, diabetes, marital status, educational level and employment status may be related to OAB.^1^,^4^,^5^ It is diagnosed clinically and proved with the help of the defining characteristics of the condition, but it ignores the use of invasive examination.^6^

The American National Overactive Bladder Evaluation study has proved that 16.5% of the participants have symptoms of OAB. It means almost 33 million adult Americans are affected.^7^ According to a survey conducted in Pakistan; OAB is found 10.8% in men and 12.8% in women.^8^ It is underestimated because a large number of patients are unable to get help as they are ignorant or embarrassed. To explain the pathophysiology of OAB, the research has proposed four theories, the autonomous theory, the afferent signaling theory, myogenic theory and the neurogenic theory. The theories try to elaborate what is alluded to as detrusor over activity.^9^,^10^ OAB might be treated through a combination of pharmacotherapy, physiotherapy and behavioral therapy. A large number of experts treat it by physiotherapy through bladder training that is considered to be a reasonable first-line therapy.^11^ Yet pharmacotherapy lets the patients of OAB get improvement earlier. Anticholinergic drugs are thought to be the major agents of pharmacotherapy such as tolterodine and oxybutynin.^12^

The prevalence of overactive bladder syndrome is rapidly evolving. As there is scarcity of existing literature in Pakistan about prevalence of OAB syndrome so this study was conducted to estimate the correct statistics of OAB. The results of this study will be helpful for the government health agencies to draw attention on this serious health issue and to reduce the burden of overactive bladder in the population of Pakistan.

## METHODS

After taking approval from ethical review board a community-based, face-to-face, cross-sectional survey was conducted with sample size 1058 recruited from different cities of Pakistan through convenience sampling technique. Written consent was taken from all participants before taking part in this study. Information sheet was provided to all participants regarding brief introduction of disease and the study being conducted. This study took three months from September to December 2020 for completion of fieldwork and data collection. Both the women and men between ages 35 to 60 years having symptoms of overactive bladder were included. People with other obvious pathological problems such as urinary tract infection, stones, prostate and tumors were excluded from the study. Data was collected by using Overactive Bladder Scoring System (OABSS) tool for prevalence and a developed questionnaire containing questions pertaining to age, gender, BMI, hypertension, diabetes, parity, smoking, constipation, Pelvic surgery, UTI and sleep to find out the associated risk factors.

Statistical Package for Social Sciences (SPSS) Version 21 was used to analyze the data. The prevalence of OAB was described in percentages whereas; the chi-square test was used to assess the association between overactive bladder and risk factors. A p-value of less than <0.05 was considered statistically significant.

### Ethical Approval

(Ref: C-31-120, Dated: Jan 31, 2020).

## RESULTS

In total OAB prevalence was 27.4% (n=289) and it increased with age. The average ages for women and men were 44.60±7.88 and 46.14±7.69 years respectively. The OAB prevalence was the lowest among the participants aged between 35-43 years old was 15.2% (n=55) while with age range 53-60 it was highest 49.6% (n=127). Between prevalence of overactive bladder and gender, gender did not have any significant relationship. It clarified that female and males might have the same chance to be affected by OAB (p> 0.06). The results proved that age was an important risk factor of OAB (p= 0.00). The BMI of the participants divided into obese, overweight, healthy and underweight. These were having an important relationship with the risk factor of OAB (p=.01). According to the results, monthly income of the participants, was an important risk factor of OAB (p= 0.01). Into three groups the number of parity was separated (nulliparous, 1 to 4 and more than 5). Data confirmed to be an important risk factor of OAB (p=0.00). Positive family history of OAB was having a significant relationship with risk factor of OAB (p=0.00). Smoking, constipation and pelvic surgery had not considered to be an important risk factor of OAB with p values (p=0.12), (p=0.90) and (p=0.27) respectively. Participant’s positive history of UTI had a significant relationship with the risk factor of OAB (p=0.00). Time of sleep was divided into four, six and eight hours sleep which showed an insignificant association with the risk factor of OAB (p= 0.16).

**Table-I:** Associated risk factors of Overactive Bladder.

Risk factor	Overactive Bladder		

Gender	Yes	No	P-Value
Male	194(25.7%)	560(74.3%)	0.06
Female	96(31.3%)	209(68.8%)	
***Age (Years)***			
35 to 43	55(15.2%)	308(84.8%)	0.00
44 to 52	107(24.4%)	332(75.6%)	
53 to 60	127(49.6%)	129(50.4%)	
***BMI (Kg/m^2^)***			
Underweight< 18.5	3(33.33%)	6(66.66%)	0.01
Healthy (18.5–24.9)	97(21.50%)	354(78.49%)	
Over weight(25.0–29.9)	169(30.39%)	387(69.60%)	
Obese(≥ 30.0)	19(46.34%)	22(53.09%)	
***Diabetic***			
Yes	130(56.52%)	100(43.47%)	0.00
No	159(19.20%)	668(80.67%)	
***Hypertension***			
Yes	91(28.34%)	230(71.65%)	0.60
No	188(25.54%)	538(73.09%)	
***Family Gross Income***			
Less than 15000	28(36.36%)	49(63.63%)	0.01
Less than 25000	79(23.72%)	254(76.27%)	
Less than 40000	181(27.97%)	466(72.02%)	
***Family History***			
Yes	138(42.85%)	184(57.14%)	0.00
No	151(20.51%)	585(79.48%)	
***Parity***			
Nulliparous	49(41.17%)	70(58.82%)	0.00
1-4	173(22.49%)	596(77.50%)	
Greater than 5	67(40.36%)	99(59.63%)	
***Smoking***			
Yes	95(31.35%)	208(68.64%)	0.12
No	193(25.59%)	561(74.40%)	
***Constipation***			
No constipation	253(27.83%)	656(72.16%)	0.90
Most of time	31(27.43%)	82(72.56%)	
All of time	5(13.88%)	31(86.11%)	
***Pelvic Surgery***			
Yes	2(66.66%)	1(33.33%)	0.27
No	287(27.20%)	768(72.79%)	
***Urinary Tract Infection***			
Yes	144(63.15%)	84(36.84%)	0.00
No	145(17.46%)	685(82.53%)	
***Sleep***			
4 hours	1(50%)	1(50%)	0.16
6 hours	13(46.42&)	15(53.57%)	
8 hours	275(26.75%)	753(73.24%)	

**Table-II:** Prevalence of Overactive Bladder.

OAB	Frequency	Percent
No Incontinence	769	72.7
Mild	189	17.9
Moderate	77	7.3
Severe	23	2.2

Total	1058	100.0

## DISCUSSION

Presently, multiple screening tools are being used to diagnose OAB at an international level. In this research, OABSS has been used and it has proved true to diagnose OAB. If the score is 1 or more, it means that the respondent has OAB.^13^

In this study, the increase of OAB and the population based survey are discussed by using the definition of International Continence Society. of 2002.^14^ According to the results of this study, 27.4% OAB is the huge increase. Many other studies also support it and estimate the increase of OAB to be between the ranges of 10.8% to 27.9%. Difference in the increase probably is due to survey methods, design of questionnaires, study populations and definition of dissimilarity of OAB.^8^,^15^

In the literature, some of the studies try to find out the risk factor of OABS. Alcohol consumption, parity, marital status, lower educational level, drug use, hypertension, obesity and advanced age may be called to be the risk factors of OABS.^1^,^4^,^16^ In this study, UTI, family history, income, parity, BMI, DM and age are connected with risk factors of OAB.

The previous experts believed that an increase of OAB was due to aging. This increase might be described by aging processes which caused bladder dysfunctions due to neurological and decreased muscle activities and changes in physical status by age-related factors, such as menopause, systemic diseases and ageing. With the increase of age, the ratio of OAB especially UI and nocturia also increased.^4^,^5^,^9^ The results of this study corroborated the same findings.

The previous studies confirmed that OAB and DM were associated with each other. Peripheral neuropathy was one of the late complications of DM. The reasons for diabetic neuropathy were metabolic derangement of the Schwann cells and damaged axonal transport resulting in impairment of nerve conduction and segmental demyelination. Therefore, peripheral neuropathy may become the reason for detrusor over activity and a significant risk factor of OAB. In the study, consistent with the literature DM was considered to be a significant risk factor of OAB.^4^,^5^,^9^,^17^

BMI was proved as a risk factor of OAB in different studies. A patient who had a BMI more than 30 kg/m²was at high risk for being victimized by OAB. Previous results proved an important relationship between OAB. Obesity might make bladder pressure more by causing detrusor instability and resulting in OAB.^7^,^9^,^16^,^18^ The results of the current study are synchronous with the previous studies.

In the literature, there were many studies which showed the association of OAB with hypertension. Vascular and hypertension risk factors resulted in increased ischemia, which led to structural changes in the bladder.^5^,^19^ The findings of this study contradict with the results of previous studies.

In many observational studies, parity was believed to be in association with OAB. Higher parity described as more than four seemed to have more significant relationship with its incidence. Hypothetical explanation which described the possible neuropathic changes by increasing the sensitivity of the detrusor muscles of the bladder during its filling sensation in pregnancy might in this way be guaranteed.^4^,^5^,^9^,^16^ The findings of current studies are in concordance with previous literature.

Studies showed that reconstructive and prior pelvic surgeries might deprive nerve supply of the bladder. The patients who had to undergo a hysterectomy might have experience of OAB symptoms after surgery due to the disruption of autonomic nerve fibers which run along the pelvic plexus.^20^ Yet, in this study, prior pelvic surgery was not found to have significance.

The studies found no proof to claim that constipation was a risk factor of OAB. But, a few studies revealed that it had significant association with OAB, and it also aggravated the symptoms.^21^

## CONCLUSION

The overall prevalence of overactive bladder was 27.4% and it does not differ by gender, hypertension, pelvic surgery, smoking, constipation and sleep while it has significant association with age, body mass index, diabetes mellitus, income, parity and urinary tract infections.

### Disclaimer

This community based survey is the part of PhD project of Mr. Sajid Rashid from Isra University.

### Author’s Contributions:

**SRA** Conceived, designed and did statistical analysis and editing of manuscript.

**SR, NB, RRK, MUK, HM & SRI:** Did data collection and manuscript writing.

**SRA & NB:** Did final review and approval of manuscript.

## References

[1] Pizzol D, Demurtas J, Celotto S, Maggi S, Smith L, Angiolelli G Urinary incontinence and quality of life:a systematic review and meta-analysis. Aging Clin Experi Res.

[2] Soler R, Averbeck MA, Koyama MA, Gomes CM (2019). Impact of LUTS on treatment-related behaviors and quality of life:A population-based study in Brazil. Neurourol Urodynamics.

[3] Ho L-Y, Chu PS-K, Consigliere DT, Zainuddin ZM, Bolong D, Chan C-K (2018). Symptom prevalence, bother, and treatment satisfaction in men with lower urinary tract symptoms in Southeast Asia:A multinational, cross-sectional survey. World J Urol.

[4] Jokhio A, Rizvi R, Rizvi J, MacArthur C (2013). Urinary incontinence in women in rural Pakistan:prevalence, severity, associated factors and impact on life. BJOG.

[5] Zhu J, Hu X, Dong X, Li L (2019). Associations between risk factors and overactive bladder:A Meta-analysis. Female Pelvic Med Reconstr Surg.

[6] Grinstein E, Gluck O, Digesu A, Deval B (2020). Update on non-invasive treatment for female overactive bladder. J Gynecol Obstet Human Reprod.

[7] Leron E, Weintraub AY, Mastrolia SA, Schwarzman P (2017). Overactive bladder syndrome:evaluation and management. Curr Urol.

[8] Salman M, Khan J, Khan A, Sulaiman S, Aslam Z, Asif N (2019). Prevalence and predictors of lower urinary tract symptoms in Pakistani men:A cross-sectional study. J Clin Urol.

[9] Wright HC, Brown ET (2018). Shared Pathophysiology of Detrusor Overactivity and Detrusor Underactivity. Current Bladder Dysfunction Reports.

[10] Averbeck MA, Goldman HB (2019). Pathophysiology of Overactive Bladder. Contemporary Pharmacotherapy of Overactive Bladder:Springer.

[11] Wolz-Beck M, Reisenauer C, Kolenic GE, Hahn S, Brucker SY, Huebner M (2017). Physiotherapy and behavior therapy for the treatment of overactive bladder syndrome:A prospective cohort study. Arch Gynecol Obstet.

[12] Hsu FC, Weeks CE, Selph SS, Blazina I, Holmes RS, McDonagh MS (2019). Updating the evidence on drugs to treat overactive bladder:a systematic review. Int Urogynecol J.

[13] Homma Y, Yoshida M, Seki N, Yokoyama O, Kakizaki H, Gotoh M (2006). Symptom assessment tool for overactive bladder syndrome—overactive bladder symptom score. Urology.

[14] Abrams P, Cardozo L, Fall M, Griffiths D, Rosier P, Ulmsten U (2003). The standardisation of terminology in lower urinary tract function:report from the standardisation sub-committee of the International Continence Society. Urology.

[15] Chuang YC, Liu SP, Lee KS, Liao L, Wang J, Yoo TK (2019). Prevalence of overactive bladder in China, Taiwan and South Korea:Results from a cross-sectional, population-based study. LUTS:Lower Urinary Tract Symptoms.

[16] Irer B, Sen V, Bozkurt O, Demir O, Esen A (2018). Prevalence and Possible Risk Factors of Overactive Bladder Symptoms in Women Living in the City of Izmir. J Urolog Sur.

[17] Narayanamurthy V, Slopnick EA, Sheyn DD, Bukavina L, Mishra K, Hijaz AK (2019). Overactive bladder in diabetes mellitus. Curr Bladder Dysfunction Rep.

[18] Lai HH, Helmuth ME, Smith AR, Wiseman JB, Gillespie BW, Kirkali Z (2019). Relationship between central obesity, general obesity, overactive bladder syndrome and urinary incontinence among male and female patients seeking care for their lower urinary tract symptoms. Urology.

[19] Torimoto K, Matsumoto Y, Gotoh D, Morizawa Y, Miyake M, Samma S (2018). Overactive bladder induces transient hypertension. BMC Res Notes.

[20] Baron MG, Quicray M, Cornu JN (2017). Urinary incontinence after hysterectomy. Am J Obstet Gynecol.

[21] Maeda T, Tomita M, Nakazawa A, Sakai G, Funakoshi S, Komatsuda A (2017). Female functional constipation is associated with overactive bladder symptoms and urinary incontinence. Biomed Res Int.

